# Supplementary figures and images for: A natural single nucleotide mutation in the small regulatory RNA ArcZ of Dickeya solani switches off the antimicrobial activities against yeast and bacteria
Source: PLoS Genet. 2023 Apr 27;19(4):e1010725. doi: 10.1371/journal.pgen.1010725 (PMC10168573; doi:10.1371/journal.pgen.1010725)

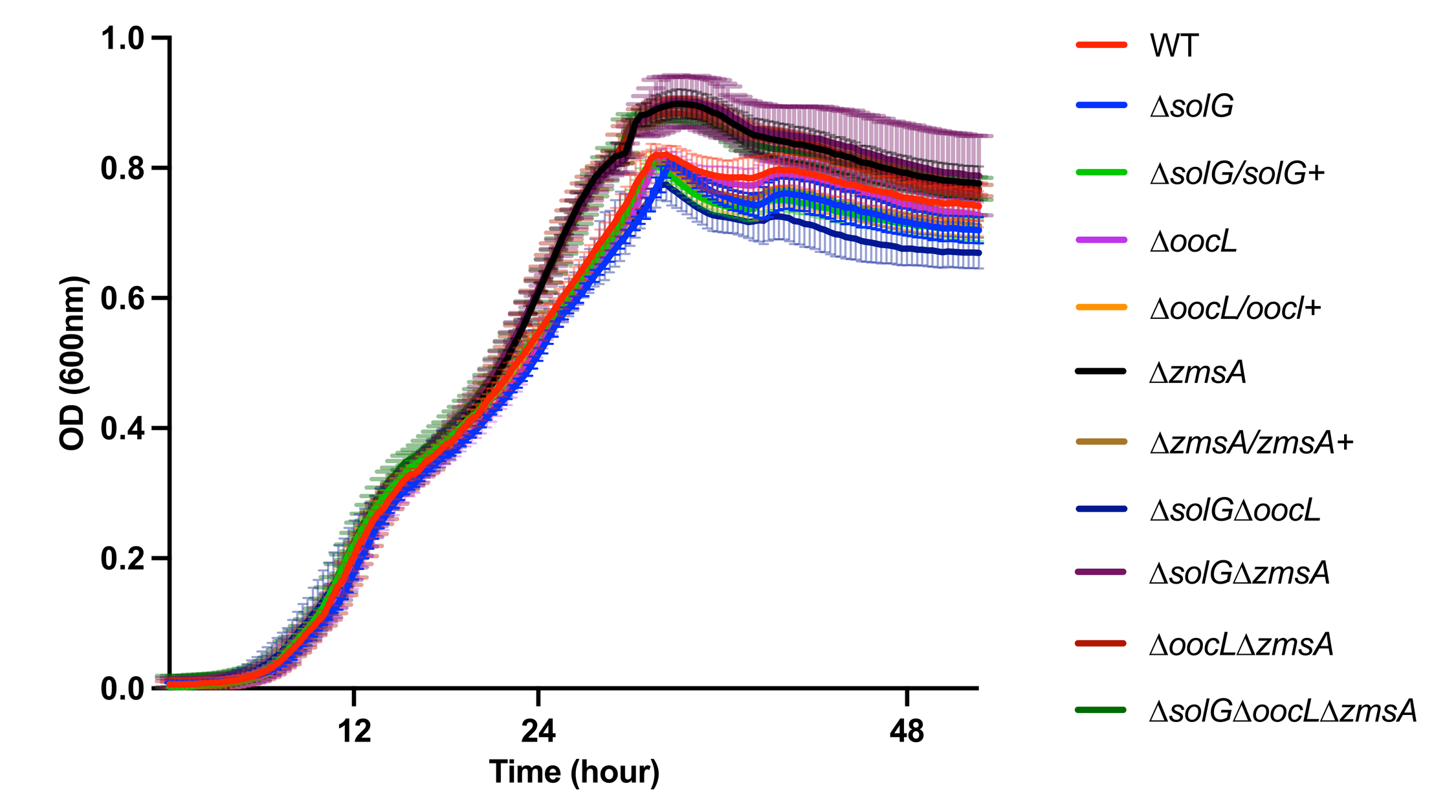

Supplement: S1 Fig — A 96-well plate containing M63 medium with 1% sucrose was inoculated with the strains used in this study at an OD of 0.06. The growth of each strain was determined by measuring OD600 every 20 minutes during 2 days in a TECAN device. The different strains showed similar overall growth, except for the light grey points where a low but significant fitness gain was observed for the mutants Δzms, Δsol Δzms, Δooc Δzms and Δsol Δooc Δzms (Mann-Whitney test, p-value<0.05). (TIF) [file pgen.1010725.s001.tif]

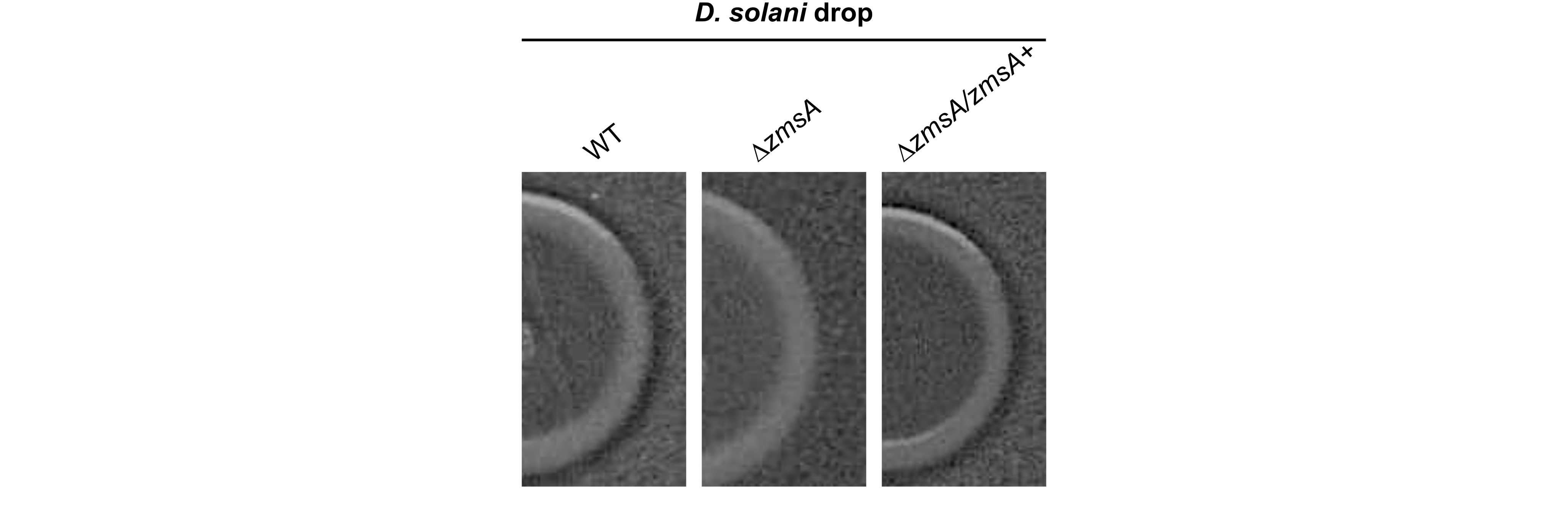

Supplement: S2 Fig — Bioassay plates were prepared by mixing E. coli culture with melted LB agar as described in the experimental procedures. 5 μL of bacterial culture at OD600nm = 2 of D. solani D s0432-1 (WT) or derivatives were spotted onto the plate and incubated at 30°C during 48 h. A slight inhibition zone was observed except with the Δzms mutant. All experiments were carried in 4 replicates. (TIF) [file pgen.1010725.s002.tif]

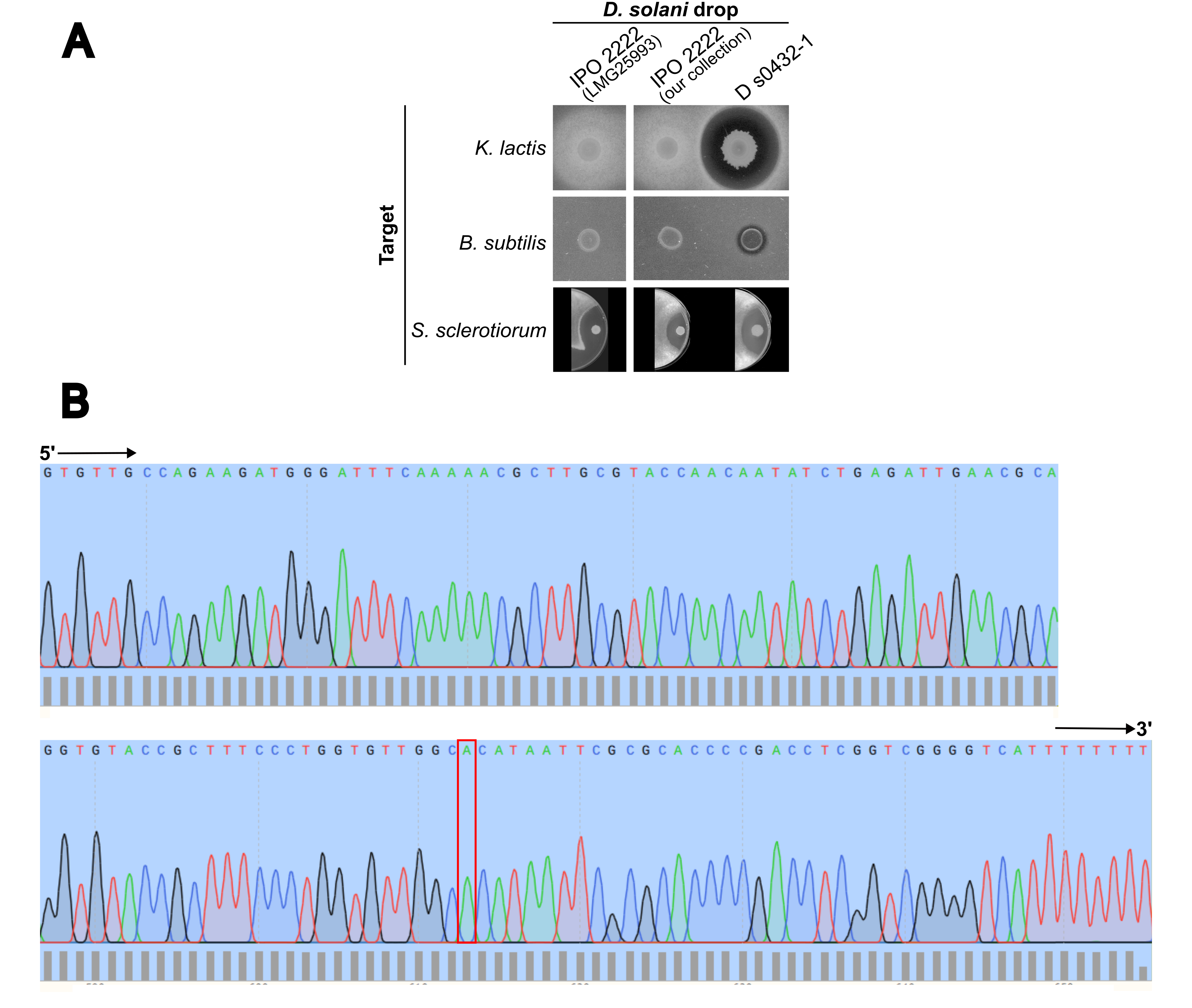

Supplement: S3 Fig — (A) Inhibition assay of K. lactis, B. subtilis and S. sclerotiorum by WT D. solani IPO 2222 LMG 25993 from the BCCM collection, compared to the strains used in this study. (B) Sanger sequencing results of arcZ of D. solani IPO 2222 (LMG 25993). The mutation G90A is highlighted by a red frame. (TIF) [file pgen.1010725.s003.tif]

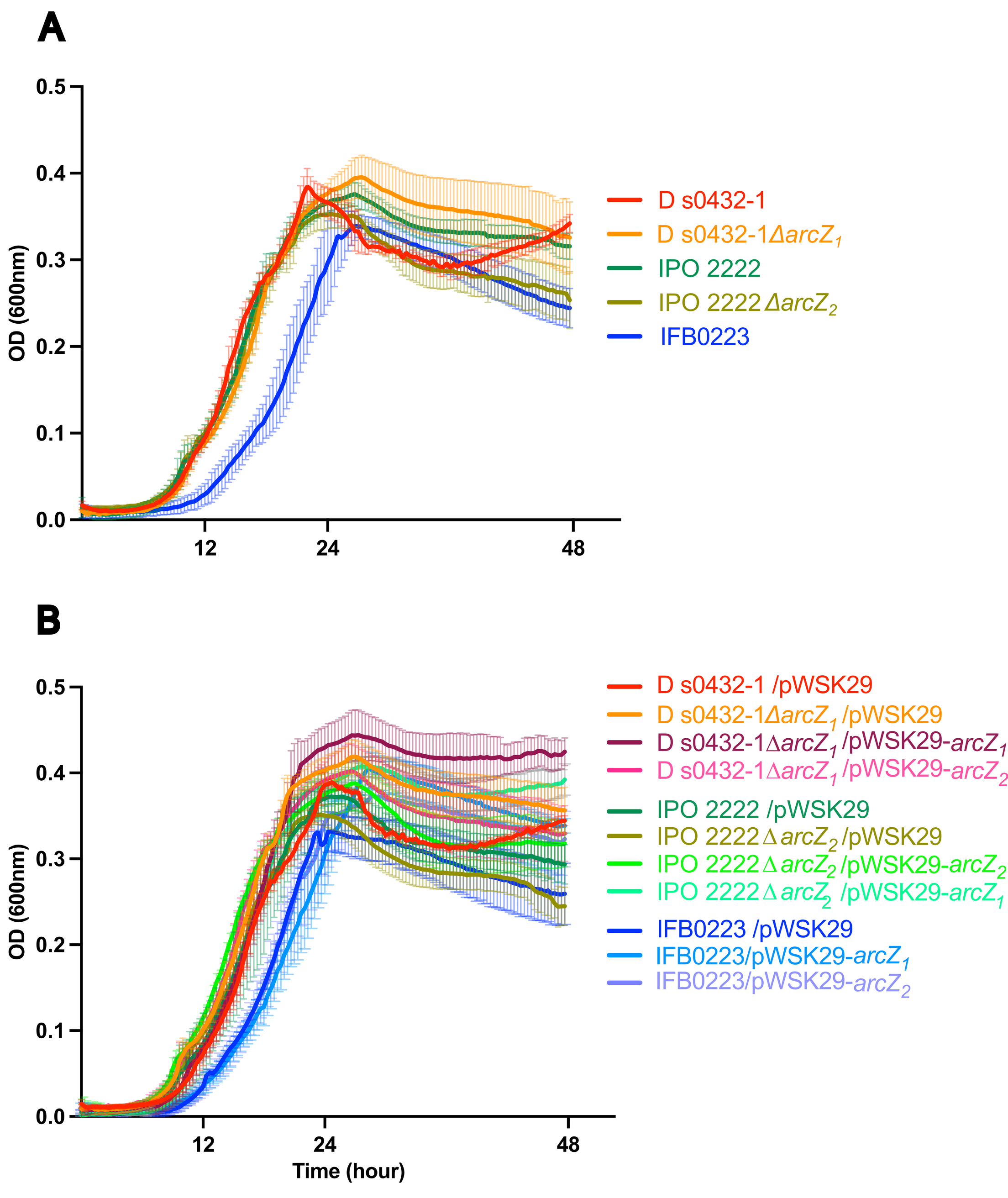

Supplement: S4 Fig — (A) Growth curves of D s0432-1, IPO 2222, their respective ΔarcZ mutants and IFB0223. The latter (in grey) has a slight growth delay (Mann-Whitney test, p-value<0.05). (B) Growth curves of strains used in complementation and heterologous expression tests. The plasmids do not cause any growth defect in D s0432-1, IPO 2222 and ΔarcZ mutants. IFB0223 derivatives containing a plasmid (in blue) have a growth delay already observed in the strain without plasmid (Mann-Whitney test, p-value<0.05). All experiments were performed in 4 biological replicates. (TIF) [file pgen.1010725.s004.tif]

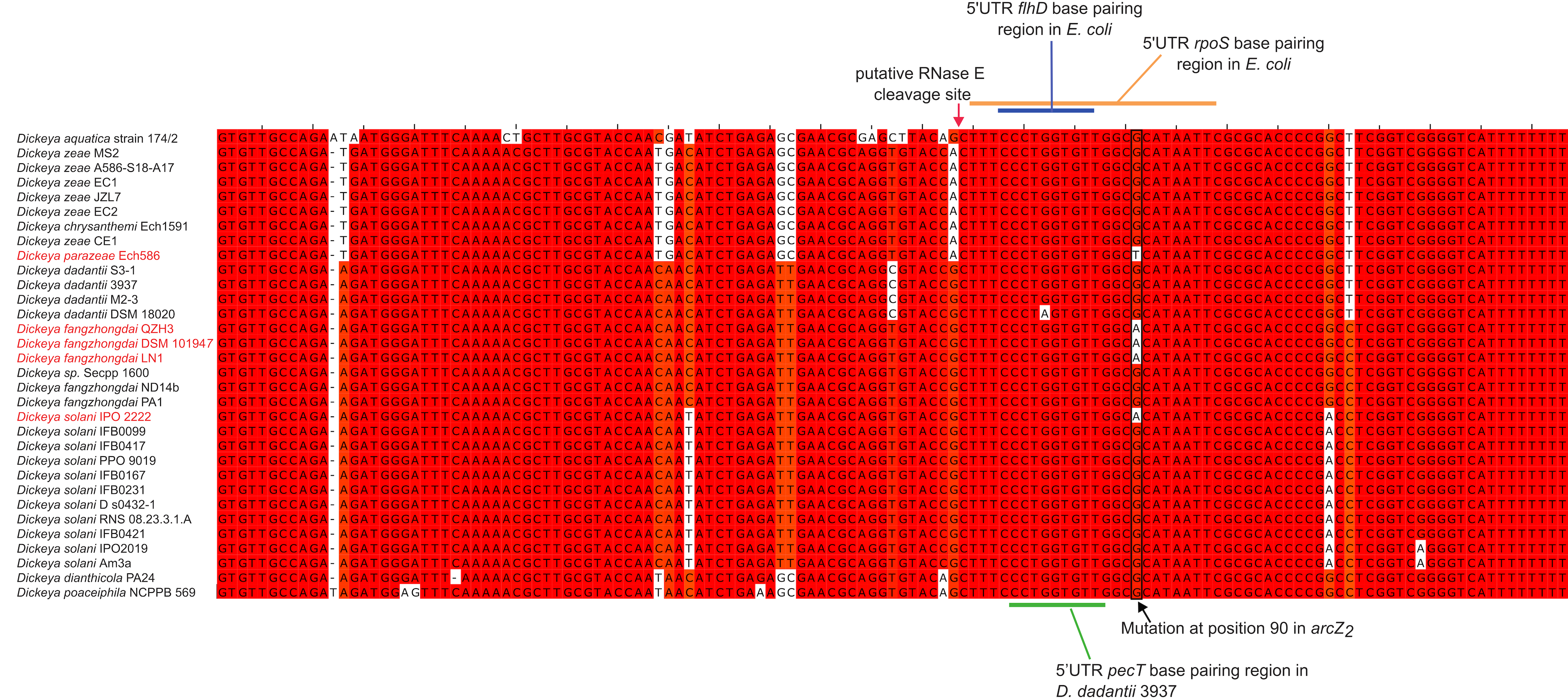

Supplement: S5 Fig — The arcZ DNA sequences were retrieved by running a BlastN on the NCBI database using the arcZ sequence of D. solani D s0432-1 as query. Search was limited to the Dickeya genomes. Then, the Dickeya arcZ sequences were aligned with E. coli MG1655 arcZ by using Jalview [50] and Muscle [51]. Known regions of interactions with the pecT, flhD and rpoS 5’UTR mRNA in D. dadantii and E. coli are indicated. (TIF) [file pgen.1010725.s005.tif]
