# Supplementary material for: A natural single nucleotide mutation in the small regulatory RNA ArcZ of Dickeya solani switches off the antimicrobial activities against yeast and bacteria
Source: PLoS Genet. 2023 Apr 27;19(4):e1010725. doi: 10.1371/journal.pgen.1010725 (PMC10168573; doi:10.1371/journal.pgen.1010725)
Supplement: S4 Table — (DOCX) [file pgen.1010725.s010.docx]

**Table S4: Bacterial strains and plasmids**

| Bacterial strain and plasmid | Description | Source |
| --- | --- | --- |
| **Strains** |  |  |
| *Escherichia coli* K12 |  |  |
| DH5α | *supE44 lacU169 (*Φ*80lacZ*∆ M15) *hsdR17 (rK mK ) recA1 endA1 gyrA96 thi-1 relA1* | Laboratory collection |
| DH5α λpir | λpir phage lysogen of DH5α | Laboratory collection |
| MFD*pir* | *RP4-2-Tc::(∆Mu1::aac(3)IV-∆aphA-·∆nic35-∆Mu2::zeo) ∆dapA::erm-pir) ∆recA* | [1] |
| MG1655 | *F^–^ λ^–^ ilvG^–^ rfb-50 rph-1* | Laboratory collection |
|  |  |  |
| *Dickeya solani* D s0432-1 |  |  |
| DS49 | *D. solani* D s0432-1 WT | Laboratory collection |
| DS50 | DS49 spontaneous Nal^R^ resistant clone | This study |
| DS58 | DS49 *glmS::Tn7-gent*, Nal^R^ Gm^R^ | This study |
| DS68 | DS58 ∆*ssmG* (∆BJD21_RS20030 of cluster A) | This study |
| DS69 | DS58 ∆*oocL* (∆BJD21_RS15005 of the oocydin cluster B) | This study |
| DS70 | DS58 ∆*zmsA* (∆BJD21_RS05130 of the zeamine cluster C) | This study |
| DS425 | DS58 ∆*ssmG* ∆*oocL* | This study |
| DS426 | DS58 ∆*ssmG* ∆*zmsA* | This study |
| DS427 | DS58 ∆*oocL* ∆*zmsA* | This study |
| DS352 | DS58 ∆*ssmG* ∆*oocL ∆zmsA* (named ∆3 mutant) | This study |
| DS354 | DS49 *D. solani* D s0432-1 ∆*arcZ_1_* | This study |
| DS428 | DS58 ∆*ssmG*/*ssmG^+^* revertant | This study |
| DS429 | DS58 ∆*zmsA/zmsA^+^* revertant | This study |
| DS466 | DS58 ∆*oocL*/*oocL^+^* revertant | This study |
|  |  |  |
|  |  |  |
| *Dickeya solani* IPO2222 |  |  |
| DS45 | *D. solani* IPO2222 WT | [2] |
| DS353 | DS45 *D. solani* IPO2222 ∆*arcZ_2_* | This study |
| DS486 | *D. solani* IPO2222 WT | Strain LMG25993 of the BCCM collection |
|  |  |  |
| **Plasmids** |  |  |
| pRE112 | Suicide vector for allelic exchange, Cm^R^, *sacB*, *oriT* RP4, *ori*R6K | [3] |
| pTn7-M | Km^R^ Gm^R^, *ori R6K*,*Tn7L* and *Tn7R* extremities, standard multiple cloning site, *oriT* RP4 | [4] |
| pTNS3 | Ap^R^, *ori R6K*,*TnsABCD* operon, *oriT* RP4 | [5] |
| pGLR2 | Kan^R^, *ori RK2*, *gfp-luxCDABE* reporter system | [6] |
| pSEVA421 | Sm^R^, *ori RK2, oriT* | [7] |
|  |  |  |
| pEGL159 | pRE112-∆*ssmG* (∆BJD21_RS20030), Cm^R^ | This study |
| pEGL160 | pRE112-∆*oocL* (∆BJD21_ RS15005), Cm^R^ | This study |
| pEGL161 | pRE112-∆*zmsA* (∆BJD21_ RS05130), Cm^R^ | This study |
| pEGL325 | pRE112-*ssmG*^+^, Cm^R^ | This study |
| pEGL326 | pRE112-*oocL^+^*, Cm^R^ | This study |
| pEGL327 | pRE112-*zmsA^+^*, Cm^R^ | This study |
| pEGL302 | pRE112-∆*arcZ*, Cm^R^ | This study |
|  |  |  |
| pWSK29 | Amp^R^, pSC101 ori, lacZp expression vector | [8] |
| pEGL332 | pWSK29-oriT | This study |
| pEGL333 | pWSK29-oriT-ArcZ_2_ | This study |
| pEGL334 | pWSK29-oriT-ArcZ_1_ | This study |
|  |  |  |
| pEGL385 | pSEVA421-prom-*sol-gfp-luxCDABE* | This study |
| pEGL386 | pSEVA421-prom-*ooc-gfp-luxCDABE* | This study |
| pEGL387 | pSEVA421-prom-*zms-gfp-luxCDABE* | This study |

1. Ferrières L, Hémery G, Nham T, Guérout A-M, Mazel D, Beloin C, et al. Silent Mischief: Bacteriophage Mu Insertions Contaminate Products of Escherichia coli Random Mutagenesis Performed Using Suicidal Transposon Delivery Plasmids Mobilized by Broad-Host-Range RP4 Conjugative Machinery. J Bacteriol. 2010;192: 6418–6427. doi:10.1128/JB.00621-10

2. Khayi S, Blin P, Chong TM, Chan K-G, Faure D. Complete genome anatomy of the emerging potato pathogen Dickeya solani type strain IPO 2222(T). Stand Genomic Sci. 2016;11: 87. doi:10.1186/s40793-016-0208-0

3. Edwards RA, Keller LH, Schifferli DM. Improved allelic exchange vectors and their use to analyze 987P fimbria gene expression. Gene. 1998;207: 149–157.

4. Zobel S, Benedetti I, Eisenbach L, de Lorenzo V, Wierckx N, Blank LM. Tn7-Based Device for Calibrated Heterologous Gene Expression in Pseudomonas putida. ACS synthetic biology. 2015;4: 1341–1351. doi:10.1021/acssynbio.5b00058

5. Choi K-H, Mima T, Casart Y, Rholl D, Kumar A, Beacham IR, et al. Genetic tools for select-agent-compliant manipulation of Burkholderia pseudomallei. Appl Environ Microbiol. 2008;74: 1064–1075. doi:10.1128/AEM.02430-07

6. Benedetti IM, de Lorenzo V, Silva-Rocha R. Quantitative, non-disruptive monitoring of transcription in single cells with a broad-host range GFP-luxCDABE dual reporter system. PLoS ONE. 2012;7: e52000. doi:10.1371/journal.pone.0052000

7. Silva-Rocha R, Martínez-García E, Calles B, Chavarría M, Arce-Rodríguez A, de las Heras A, et al. The Standard European Vector Architecture (SEVA): a coherent platform for the analysis and deployment of complex prokaryotic phenotypes. Nucleic Acids Research. 2013;41: D666–D675. doi:10.1093/nar/gks1119

8. Wang RF, Kushner SR. Construction of versatile low-copy-number vectors for cloning, sequencing and gene expression in Escherichia coli. Gene. 1991;100: 195–199.
